# From Occasional Choices to Inevitable Musts: A Computational Model of Nicotine Addiction

**DOI:** 10.1155/2012/817485

**Published:** 2012-11-20

**Authors:** Selin Metin, N. Serap Sengor

**Affiliations:** Electrical and Electronic Engineering Faculty, Istanbul Technical University, Maslak, Istanbul 34469, Turkey

## Abstract

Although, there are considerable works on the neural mechanisms of reward-based learning and decision making, and most of them mention that addiction can be explained by malfunctioning in these cognitive processes, there are very few computational models. This paper focuses on nicotine addiction, and a computational model for nicotine addiction is proposed based on the neurophysiological basis of addiction. The model compromises different levels ranging from molecular basis to systems level, and it demonstrates three different possible behavioral patterns which are addict, nonaddict, and indecisive. The dynamical behavior of the proposed model is investigated with tools used in analyzing nonlinear dynamical systems, and the relation between the behavioral patterns and the dynamics of the system is discussed.

## 1. Introduction

The value of an experience or an action is imposed by the reward gained afterwards. An action inducing a greater reward is sensed as a superior action, and thus the successive occurrences of this type of actions are rewarded more frequently [1]. In the case of addiction, the abusive substance (nicotine, drugs, etc.) has a value greater than other forms of reward imposing actions. Some persistent modifications in the synaptic plasticity in the neural subsystems of the brain are believed to lead to addiction; thus, it is claimed that addiction is a disorder in the mesolimbic system which develops by the modification of responses to rewarding actions [1–4]. Mislead by the overemphasized reward perception, the addicts compulsively seek for the substance they are addicted to. Since the reward mechanism has been persistently changed, addicts usually cannot be completely cured, and they often relapse into drug use after treatment [2].

Considering the negative social and physical impacts of addiction, any attempt to understand the neural mechanisms underlying this phenomenon is valuable. So, in this study, a computational model of addiction is given. We propose a model for nicotine addiction which is composed of a cortico-striato-thalamic action selection circuit and a dopamine signaling unit operating according to reinforcement learning. The proposed model is based on the interaction of neural structures known to be taking role in addiction and the effect of neurotransmitters on these structures. The proposed model focuses on the neurophysiological basis of addiction and the results obtained by the model show that the model is capable of demonstrating different behavioral patterns. (See the Supplementary Material containing the source codes available online at doi:10.1155/2012/817485 obtained from http://www.selinmetin.info/).

Since neurophysiological aspects are considered, we concentrated on the interaction between limbic and cortical structures. These structures have been considered not only for addiction but also in explaining a large spectrum of cognitive processes *‎*[3–5]. The two main behavioral approaches used to explain addiction are the opponent-process theory and reward-related learning *‎*[6–8]. Using reinforcement learning theory, addiction is explained as the cumulative result obtained by the administration of a drug as a positive reinforcer *‎*[8–10]. The opponent-process theory of motivation *‎*[6] is used to explain the conditioning principles leading to pleasurable and compulsive activity. According to opponent-process theory, emotions are paired, and when one emotion in a pair is experienced, the other is suppressed. In *‎*[11], these two approaches are considered together in deriving a computational model for nicotine addiction. Our model also combines these two approaches to design a dynamical system of addiction development. The proposed dynamical model fulfills the expectation from any computational model of a biological process; it gives a formal approach which can be improved further to understand the mechanisms behind a biological process rather than just mimicking the outcomes of the process.

Our aim in this paper is to support the idea that addiction development is a form of goal-directed behavior, and the interaction of corticostriatothalamic action selection loops has an important role in the addiction development process. We hypothesize that nicotine addiction is a transition from impulsive behavior to compulsive behavior developed through reinforcement learning. We propose an initial model of addiction based on the interactions of limbic and cortical structures. The model is influenced by a computational model of goal-directed behavior *‎*[12] and nicotine addiction *‎*[11]. Although in *‎*[11] an action selection module using winner-takes-all mechanism is presented, the action selection part of our model is capable of revealing reinforcement learning elements. As our aim is to give a formal approach, we use dynamical systems theory in modeling nicotine addiction behavior. Through reinforcement learning, the dynamical system modeling action selection (A-S) determines the fixed points it settles down. These fixed points which change with evaluation of choices by reinforcement learning correspond to behavioral choices made while developing an addiction. This behavior of A-S module is explained using bifurcation diagrams obtained by XPPAUT, while the addiction process is simulated with an in-house built m-file in MATLAB. So, a formal approach based on nonlinear dynamical system evaluation with time and parameter change is proposed to model a biological process.

The results and the model equations were partly published in *‎*[13, 14]. However, all the aspects of our model, including the background neurophysiology and supporting material from the literature, are presented in a compact form for the first time in this paper.

A short summary on the computational models related with cognitive processes claimed to have role in addiction is given in Section 2. This summary is given to give an idea about what is the state of the art in computational modeling and to build a necessary background to evaluate the model proposed here in connection to related work in the literature. In Section 3, neural and behavioral aspects of addiction are explained as the proposed model aims to give a formal approach in comprehending both of these aspects of addiction. In Section 4, the proposed model is introduced, and then implementation of it is explained in detail so that the results can be reproduced. These implementations are explained in two parts covering both the analysis of dynamics of A-S module and realization of reinforcement learning to adapt the choices of A-S module. In Section 5, the simulation results obtained by the model are given and discussed. In Section 6, a discussion of the model comparing it with the models discussed in Section 2 is given.

## 2. Computational Models of Addiction

Mathematical models of drug addiction can be grouped into three main titles: quantitative pharmacological models studying the effects of dopamine (DA) in reward mechanism, computational models studying with the effects of DA in reinforcement learning and action selection, and neurodynamical models studying opponent-processes and dopaminergic system of the brain *‎*[15]. 

Reinforcement learning can be briefly described as learning from the experiences while interacting with the environment to achieve a goal. The learner or decision maker continuously interacts the environment to select an action and the environment responds to the actions presenting new situations to the learner *‎*[16]. The opponent-process theory [6] claims that the conditions that lead an individual to make choices do not depend only on the rewarding qualities of the actions but also on the general experience and the situation of the individual. Whenever a pleasing stimulus is experienced, an immediate liking response develops and quickly makes a climax. If the stimulus remains stable, this response slowly fades to reach an equilibrium state. When the stimulus disappears, this time a negative response develops to make a negative climax. These two responses are called opponent-processes. 

Here, we will summarize the state of the art in computational modeling of reinforcement learning and opponent-processes and especially mention the models related to addiction.

### 2.1. Models of Opponent-Process and Reinforcement Learning

Pharmacological models can be subgrouped into positive and negative feedback models. Positive feedback models claim that drug consumption is not a goal-directed behavior, but an automatically stimulated process when brain drug level decreases. On the other hand, negative feedback models hypothesize that drug consumption is a form of goal-directed behavior. The downside of both approaches is that they cannot bring an explanation of how addiction develops. A good example of computational models is given in *‎*[17], and this model uses temporal difference reinforcement learning principle to calculate and update the value of each situation. However, it lacks explaining how the reward function takes part in the process of learning.

Opponent-processes are claimed to be important for emotional systems in the brain. They give insight especially in explaining the evaluation of experiences and calculation of reward expectations. Several models have been proposed to explain the role of opponent-processes in addiction and goal-directed behavior. Here, we will summarize some of these models which are important either for their approaches to the development of addiction or the overall integrity in explaining the behavior of addiction.

Grossberg and Gutowski *‎*[18] use gated dipoles to explain opponent-processes in decision making. Their hypothesis, the affective balance theory, explains the decision making process under risky situations using psychophysiological mechanisms. The output of a single neuron cell is determined by the input and the dynamically changing equation of a neurotransmitter. The multiplication of a constant input signal and a slowly changing neurotransmitter concentration result in a rapid increase phase, a habituation phase, and a rapid decrease phase in the output signal. When used as a two-neuron complex, they call this minimal neural network structure a gated dipole. They use the gated dipole opponent-process to model how the onset or the offset of a reinforcer can be linked to a conditioned stimulus (CS). The resultant output graph of a gated dipole is quite similar with Solomon and Corbit's [6] description of opponent-processes. However, there are some major differences. *‎*[6] explains opponent-processes by the subtraction of two antagonistic processes. Since *‎*[6] approaches the subject from a behavioral and psychological perspective and does not try to give a dynamical or even a mathematical model, the different time scales of the two antagonistic processes and how the latter one is triggered by the former one are not clearly explained. Grossberg's model gives an insight to this problem by hypothesizing about a slowly habituating and tonically aroused gating neurotransmitter signal.

In *‎*[19], a specialized gated dipole circuit, the READ circuit, is developed to explain phenomena like secondary inhibitory conditioning. The READ circuit combines the opponent-processing and associative learning mechanisms. Since it is based on the gated dipole structure, the READ circuit operates using processes acting on three main time scales: a fast activation time scale, a slower habituation time scale, and an even slower conditioning time scale. These processes are linked together by nonlinear feedback interactions. The gated dipole circuits are combined with competitive interactions that choose between motivational drive representations using a winner-take-all mechanism. In *‎*[19], Grossberg and Schmajuk designed and compared several specialized gated dipole circuits to analyze behavioral data and related brain signals. They used these circuits to examine the motivation, conditioning, attention, fast information, and slow learning mechanisms in the brain, and the role of short-term and long-term memory in these mechanisms.

In *‎*[20], Daw et al. use dopamine (DA) and serotonin as the two neurotransmitters triggering the opponent-processes of appetitive and aversive systems. They use DA as the main element for coding the error signal in temporal difference reinforcement learning. Additionally, they claim that serotonin is responsible for the expression of long-term predictions and aversive stimuli in the temporal difference reinforcement learning (TDRL) error signal. They try to model both the short- and long-term aspects of opponency by using the interactions of phasic DA and tonic serotonin signals which are responsible for reporting the average reward prediction. This reward formulation provides a computational explanation from the scope of reinforcement learning for the opponent-process model given by Solomon et al. This model simplifies the average reward rate as a static function and considers only two time scales taking part in the opponency mechanism.

Dezfouli et al. *‎*[21] enhance Daw's [22] temporal difference (TD) model of DA system. Daw's model *‎*[22] is based on average reward computed as an exponentially weighted moving average of experienced rewards. Due to the alteration of the brain reward system caused by long-term drug usage, the base reward level against which rewards are measured is abnormally increased. This increase is caused by a decrease in DA functioning. Basal reward level is considered as an internal variable in the decision-making system, and it is claimed to be corresponding to the average reward. Basal reward level appears as a negative term in the calculation of the error signal and thus affects the decision-making system. The growth of the estimated value of the drug stops when error signal *δ*_*t*_ < 0. This model has some similarities with Gutkin et al.'s model *‎*[11], but it does not take into account the drug dose. This model does not contain some of the brain structures like amygdala, prefrontal cortex (PFC), and ventral pallidum which are important in addiction development.

Guthrie and colleagues developed a network model of the striatal direct DA pathway for action selection in *‎*[23]. This network can sequentially learn a task which is dependent on the basal ganglia. By manipulating the phasic and tonic levels of DA, the model can demonstrate the symptoms of Parkinson's disease in humans. The authors claim that the results show that the dysfunctions in Parkinson's disease are caused by a combination of decreased tonic DA level and responsiveness of the phasic DA signal to reward and punishment.

### 2.2. Computational Models of Nicotine Addiction

A computational approach to model the role of opponent-process in nicotine addiction is developed by Gutkin and colleagues *‎*[11]. They model the chemical activity of the nicotinic acetyl choline receptors (nAchRs) in three different time scales according to the concentration of DA in the environment: activation, upregulation, and the long-term opponency. Their molecular approach is different than the mechanistic approach of Grossberg and colleagues *‎*[18, 19], and they cannot define the scope of the time scales explicitly. However, theirs is a complementary research to explain this psychological-molecular-neurophysiological phenomenon.

Although it is not a limbic system model, it is worth mentioning the work of Graupner and Gutkin [24, 25] here. They developed a mean field circuit model of DA secretion from the ventral tegmental area (VTA). They take into account the average activity of DA and gamma-aminobutyric acid (GABA) neurons in the VTA under acetyl choline (Ach) and glutamate (Glu) input and by considering the nAchR activation/desensitization according to different subtypes of nAchRs. This paper extensively summarizes the interworking of DA and GABA neurons in the VTA to secrete DA to the mesolimbic reward system.

For VTA DA cell activity, a molecular level approach which simulates the tonic firing and bursting patterns is developed in *‎*[26]. Theirs is a single-compartmental model demonstrating the qualitative behavior of dopaminergic neuronal dynamics, especially under the presence of nicotine. This model is similar to the well-known Hodgkin-Huxley model for neuron functionality, and calcium and potassium channels on the cell membrane are key elements of the model. The conductance-based approach used in this model corresponds to the pharmacological manipulations caused by the drug apamin.

Even though these two last computational models are versatile for explaining the mechanisms of DA segregation, they are not intended to be used in clarifying the role of DA in addiction. Though all these models are important milestones in computational modeling of opponent-process, reinforcement learning, and reward-related DA segregation, they do not aim to explain the mechanisms behind addiction based on the mutual role of limbic and cognitive neural circuits.

## 3. Neural and Behavioral Mechanisms of Addiction

Addiction is feeling an extraordinary high affection for a substance, and developing an addiction can be considered as a kind of behavior learning. The object of addiction is overvalued, and this overrated pleasing quality of the addictive substance causes more frequent consumption of it *‎*[1]. Directly proportional to its value, the absence of the addictive substance is perceived as a source of stress and grief. This negative affection feeling causes a compulsive seeking behavior *‎*[2]. Positive and negative affections are two opponent emotions which are closely related to each other, and any disruption in this relationship causes one of these emotions to be felt extremely, which later results in addictive behaviors.

The World Health Organization defines addiction as the dependence syndrome which is a cluster of physiological, behavioural, and cognitive phenomena in which the use of a substance or a class of substances takes on a much higher priority for a given individual than other behaviours that once had greater value. Before a person becomes addicted, there is a time period in which the neuroplasticity is reversible. Some persistent modifications in the synaptic plasticity are believed to be the main cause of addiction. Therefore, we can consider addiction as a disorder in the mesolimbic reward system of the brain, which irreversibly modifies responses to rewarding actions. Since the plasticity is lost, the (new) values of actions/substances are not learned correctly, and the values of the former actions that have lead to the loss of plasticity are stamped in. 

Due to the persistent change in the reward mechanism, addicts are usually not completely cured, and they relapse into drug use after treatment *‎*[2].

Several stages are defined to become addict in cigarette smoking by *‎*[27] as the transformation from irrelevant thoughts to occasional smoking, from occasional to chronic, and from chronic to addicted smoking. Since impulsivity is defined as a sudden irresistible, irrational desire or action resulting from a particular feeling or mental state, and compulsivity is the repetitive action that is the object of an overwhelming urge to perform an irrational act *‎*[28], addicted smoking can be considered as a transfer from impulsive behaviors to compulsive behavioral patterns. Through the process that leads to addiction, smoking is first a response to the stress caused by an individual's environment. Then, it starts to regulate the externally stimulated needs, and finally the urge to smoke cannot be suppressed since it became a reaction against internal stimulation, such as blood level of nicotine *‎*[29].

### 3.1. Neurophysiology of Opponent-Process and Reinforcement Rearning

Among the hypothesis explaining the development of addiction, reinforcement learning and associative processes have a strong ground. According to the conditioning approach, which puts reinforcement learning in the focal point, addiction is explained as the cumulative result of drug self-administration as a positive reinforcer *‎*[8–10]. The rewarding quality of an addictive substance causes increase in the frequency of the behavioral choices that lead an individual to seek more of those substances. Thus, addictive substances are positive reinforcers that maximize the measurable reward obtained for a specific type of behavior. Each time an individual decides to consume an addictive substance, the value of this action is stored in the associative memory. On the next decision moment, this stored value determines the probability of selecting that action again. 

**Figure 1:**
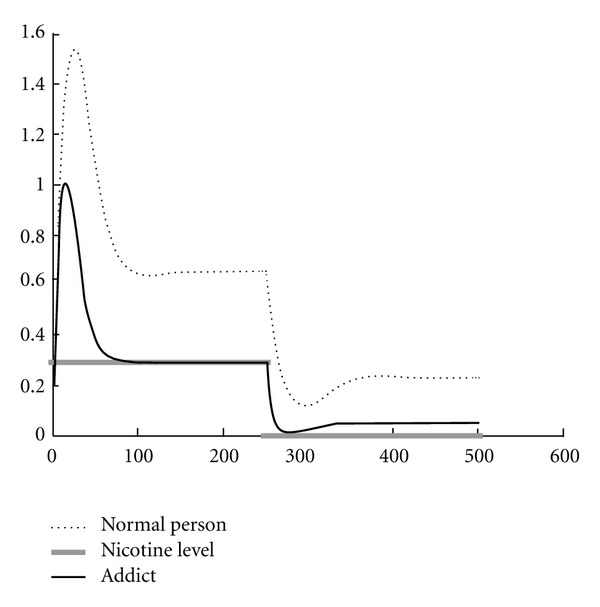
Modification of opponent-process graph with the development of addiction. The comparison of opponent-process in normal people (dotted line) versus an addict (straight line) for the reaction of nAchRs against the blood nicotine level (grey line). Related computational explanation is in Appendix A.

The behavioral aspects of addiction are explained by the opponent-process theory. The opponent-process theory of motivation proposed by Solomon and Corbit *‎*[6] is used to explain the conditioning principles leading to pleasurable and compulsive activity. The opponent-process theory claims that the situations that lead an individual to make a choice are not only the rewarding qualities of the substances, but also the general experience and the actual situation of the individual. According to this model, emotions are paired, and when one emotion in a pair is experienced, the other is suppressed. When a stimulus, either pleasing or annoying, is experienced, a primary reaction develops, quickly reaches a peak point and settles at a countenance state. When the stimulus disappears from the environment, the primary reaction also disappears and a secondary reaction develops in the opposite direction of the first one. The secondary reaction also reaches a climax very quickly and disappears as time passes. These pairs are called A- and B-processes. Repeated experience of opponent emotion pairs strengthens them, while experiencing them less weakens the opponent-process. Especially the B-process is amplified directly proportional to its development frequency. The B-process is dependent on the A-process because it is indirectly triggered by the A-process. For healthy individuals, opponent-processes are experienced almost equally (such as fear and relaxation afterwards), but for addicts, the B-process lasts longer and has a greater magnitude (Figure 1) *‎*[6, 7, 30].

The change in behavior which is explained previously has a neurophysiological background. The withdrawal symptoms have the same effect on an addict's mesolimbic system. When addiction has developed, the reward perception level of the brain is modified so the magnitudes of A- and B-processes will decrease. The neutral level, which shows the amount of addictive substance in the environment, will also decrease. We can picture this situation for a cigarette smoker. At the beginning, the individual has no positive or negative thoughts against nicotine. After smoking for some time, the action of smoking starts to feel good, and when the cigarette is burnt completely, its absence is experienced as a negative affection. These positive and negative moods form the mechanism of opponent-process. In our model, the opponent-process shows itself in the reaction of the nicotinic acetylcholine receptors (nAchRs) to the blood nicotine level.

Allostasis is the modification of the organic systems to adapt their homeostatic equilibrium state to a new state in order to answer chronic needs [7, 30]. In other words, allostasis is maintaining the stability of the organism when modifications continue. The change in the perception of the rewarding qualities of the abusive substance is an allostatic adjustment, and the stability that should be preserved is the stability of the reward function. By examining the motivation systems in the brain, it can be stated that the A-process is countered by the homeostatic modifications of the brain systems (B-process) *‎*[30]. Since the malfunctioning reward mechanism in the brain leads to addiction, the related negative emotional state increases the abusive substance consumption. Once the reward mechanism is broken, the B-processes defining the normal homeostatic limits of the reward function cannot go back within the normal limits again.

### 3.2. Molecular Basis of Nicotine Addiction

The most effective neurotransmitter in the mesolimbic reward system, and thus the addiction mechanism, is dopamine (DA). The value of the possible behavioral choices is calculated by using the outcomes of the past actions, and this value is stored in the memory. This stored information is used to predict the outcomes of future actions. DA is supposed to code the error value computed by comparing the outcome of the action and the reward gain of the prediction. This error value updates the stored information to use in future selections. Thus, DA shapes future actions in order to increase reward gain *‎*[1, 30]. The higher the reward gain for an action is, the more frequently it is repeated. DA reinforces this value-action link and has a critical role in the development of addictive behaviors *‎*[31, 32]. Adaptive changes in DA transmission cause nonassociative, long-lasting, and eventually irreversible modifications (sensitization to DA) in the DA system, resulting in addiction [33].

**Figure 2:**
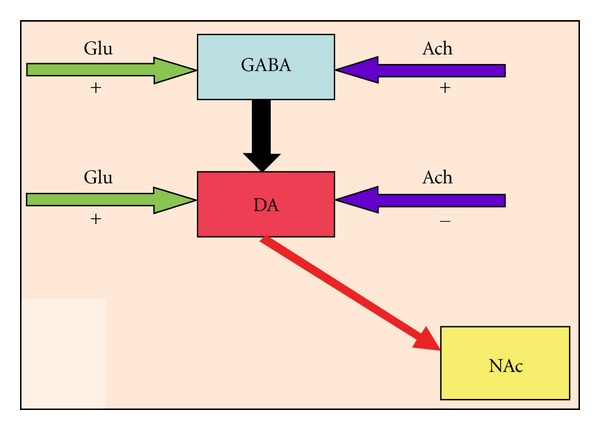
Inputs and interconnections of VTA DA and GABA neurons. Ach; acetyl choline; DA; dopamine; GABA; gamma amino-butyric acid; Glu; glutamate; NAc; nucleus accumbens.

A closer look at the neuromodulatory system of DA secretion from the ventral tegmental area (VTA) reveals two components taking part, both for the excitatory and inner dynamics: the principal excitatory inputs to the VTA are acetyl choline (Ach) from amygdala-lateral hypothalamus-pedunculopontine tegmental nucleus/laterodorsal tegmental nucleus (Am-LH-PPTg/LDTg) block and glutamate (Glu) from cortical regions (prefrontal cortex). These projections synapse on DA and gamma-aminobutyric acid (GABA) neurons in the VTA (Figure 2), modulating their activity. The main inhibitory inputs to the VTA are GABAergic and project from nucleus accumbens (NAc) and ventral pallidum (VP). Inner dynamics of the VTA is composed of the interaction of DA and GABA neurons. Ach or Glu input to GABA neurons increases their activity resulting in increased levels of GABA. On the other hand, increasing GABA activity has an inhibitory effect on DA neurons. Glu input to DA neurons at the same time with GABA neurons causes an initial increase in the DA neuron activity. However, with increasing GABA neuron activity, a steep decrease is observed. On the contrary, Ach input to DA neurons decreases their activity, having a dip level at the peak time of GABA neuron activity. When Ach and Glu are given together, an even higher increase is observed in GABA neuron activity and a biphasic response is observed in DA neuron activity. Thus, DA neuron activity first increases, having a peak at the peak time of GABA neuron activity, and then decreases very quickly, producing the well-known shape of the opponent-process.

Continuous nicotine exposure results in desensitization and causes the GABA neuron to decrease its activity dramatically in response to Ach input. The decreased inhibition from GABA neurons leads to a long-lasting excitation of the DA neurons, causing the DA neuron activity to be in a higher level than the neutral state (without tonic nicotine exposure) *‎*[24, 25, 34, 35].

Ach encodes the expectation of information for potential choices in a learned task. Thus, Ach signals the internal representation of demands in a learned task *‎*[24, 25, 36]. Glu is responsible for learning processes in the dorsal striatum-related brain substructures which take part in the behavioral decisions, namely the action selection circuitry [11, 24, 25].

With long-lasting administration of nicotine, the rewarding effect of DA diminishes because of the opponent-process. This causes a modification of the DA neuron plasticity resulting in a control gap of the ventral DA pathway for nicotine metabolism. At this point, compulsive drug seeking is established [24, 25]. These modifications in the mesocortical DA system and their glutamatergic feedback loops have stimulating effects on drug seeking behaviors and relapses into drug use [2].

### 3.3. The Neurophysiological Basis of Our Model

**Figure 3:**
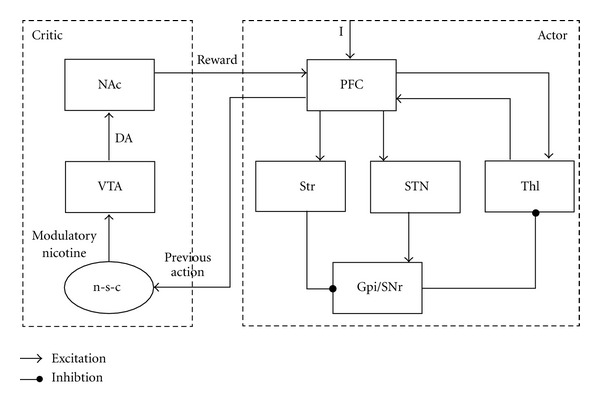
The pathways taking role in goal-directed behaviors. Actor and critic elements of reinforcement learning are shown with dotted lines. GPi/SNr: globus pallidus internus/substantia nigra pars compacta; I: external inputs; NAc: nucleus accumbens; n-s-c: opponent-processes stimulated by nicotine presence; PFC: prefrontal cortex; STN: subthalamic nucleus; Str: dorsal striatum (caudate and putamen); Thl: thalamus; VTA: ventral tegmental area.

The neurological pathways behind nicotine addiction contain several loops within the limbic substructures of the brain. We adopt the dopaminergic approach to the goal-directed behavior and development of addiction. In Figure 3, some of the pathways important in goal-directed behaviors from the scope of our model are drawn. There are two important DA secretion centers in the brain which are important for the behavioral choices based on reward evaluation *‎*[10, 37]: VTA and substantia nigra pars compacta (SNc). The DA neurons in the VTA project to the limbic forebrain (NAc, Am, hippocampus) and the prefrontal cortex (PFC). The DA neurons in the SNc project to the dorsal striatum (caudate nucleus and putamen) *‎*[1]. All of these substructures, except for the NAc, have glutamatergic excitatory connections with the VTA. The main mechanism which triggers the DA secretion from the VTA/SNc is the excitation of DA neurons in the VTA/SNc by the Am, LH, and the PPTg/LDTg block which is stimulated by the LH. On the other hand, striatum sends inhibitory signals to the VTA DA secretion system. The major glutamatergic excitatory inputs to the VTA are from the PFC and PPTg/LDTg. The GABA neurons in the VTA provide a local inhibition to DA secretion system *‎*[24, 25]. The timing differences between the excitatory and inhibitory signals cause the DA neurons in VTA/SNc to burst, resulting in sudden increases in the DA secretion or halts resulting in DA dips *‎*[37]. The glutamatergic inputs from the PFC excite the DA and GABA neurons in the VTA. Increasing GABA secretion in the VTA has inhibitory effects on the VTA DA neurons, resulting in a decrease in the DA level.

During learning, ventral striatum (NAc) firing increases, and this activity causes the representation of the environment to be stamped in. These representations are then used to calculate the reward value and the expectancy of action-situation pairs *‎*[38]. 

## 4. The Proposed Model for Nicotine Addiction

In this paper, the approach proposed in *‎*[11] for nicotine addiction is combined with the goal-directed A-S system given in *‎*[12]. The model has two parts: a DA signaling module responsible for the reinforcement learning task and an action selection (A-S) module. A-S module is a well-studied cortex-basal ganglia-thalamus dynamical system. The DA signaling module is driven by the activity of the nAchRs which are stimulated by the presence of nicotine.

DA signaling module is composed of an action evaluation part which operates based on the nicotine level and a value assignment part which calculates the rewards assigned to the performed actions and an expectation error. The DA signaling module drives the A-S loop with the representation of hedonic value of the previous actions. The proposed model (Figure 3) captures this process through reinforcement learning by adapting a parameter, namely W_r_, that denotes the effect of VTA DA signaling on action selection.

### 4.1. The Neural Structures Considered in the Model

In Figure 3, a schematic representation of our model is given. Here, dorsal action selection loop is sketched together with VTA and NAc taking part in the reward evaluation process. This action selection loop corresponds to the actor element of reinforcement learning. The globus pallidus interna/substantia nigra pars reticulate (GPi/SNr), striatum (Str), and subthalamic nucleus (STN) nuclei of the basal ganglia contribute to the action selection by calculating the value of the rewards and the error in the reward arrival times, and they pass this information to the PFC over the thalamus (Thl). This circuit guides the future actions based on the value of the current rewards. The information about the positively evaluated, that is, more efficient, actions is stored in the dorsal striatum. The dorsal striatum, particularly the caudate nucleus, is involved in social learning *‎*[10]. The PFC is the final frontier in the goal-directed behavior, as it guides the individual to decided goals and inhibits harmful actions. The motor areas in the PFC are the output of this system since they perform the chosen actions as behaviors in the individual's environment. 

NAc is important for the evaluation of positively perceived emotional situations such as reward and motivation *‎*[38]. Natural rewards and abusive drugs increase the amount of the synaptic DA in the NAc and therefore have similar effects in initiating behaviors. However, they do not increase DA transmission in the medial PFC (mPFC) where mesocortical DA neurons terminate *‎*[33]. Along with NAc, Am, hippocampus, and orbitofrontal cortex (OFC) have important roles in the evaluation of rewards and establishing memories related with rewards. The DA secretion in the NAc relates the pleasing qualities of a goal to the motivation. The DA release in the NAc, PFC, Am, and Str identifies the motivational importance and value of certain experiences. However, the DA neurons in the VTA serve as the DA resource of the ventral limbic DA subsystem. The DA neurons in the VTA are stimulated by opponent-process related modifications in the molecular level, and, hence they modulate the DA secretion effective in reward evaluation (Figure 3). The modulatory nicotine and the evaluation of previous actions by NAc is the critic element of reinforcement learning in our model.

### 4.2. Dynamics of the Action Selection Module

Dynamics of the A-S system is studied using XPPAUT *‎*[13], and all the related equations are given in Appendix B. Although the A-S system is comprised of premotor and motor parts, since these two are very similar, we study the dynamics of the premotor system. Only cortex component, *p*_pm_, is taken into account since it is the output of the premotor A-S system that drives the motor loop. Reinforcement learning is provided by changing *Wr*_pm_ parameter in the model which causes the selected actions to be modified. The effect of the modification of *Wr*_pm_ on the premotor system is demonstrated with the bifurcation diagrams. As this parameter changes through reinforcement learning, the equilibrium points of the dynamical system and their stability change along.

In order to explain explicitly what is going on during the operation of the model, the set of initial parameter values given below are considered. Before learning begins, the randomly selected weight matrices are as follows:

(1) $$\begin{array}{c} W_{r}=\left[\begin{array}{c} 0.5061\\ 0.5061 \end{array}\right],\;\;W_{c}=\left[\begin{array}{cc} 0.5410 & 0.1935\\ 0.3310 & 0.4624 \end{array}\right],\\ W_{v}=\left[\begin{array}{cc} 0.0185 & 0.0176 \end{array}\right]. \end{array}$$

Using these weights with parameters taken as *I* = 0.1, *W*_dpm_ as a 2 by 2 matrix composed of 0.5's, *λ* = 0.5, *a* = 3, the following fixed points (Table 1) are obtained for the premotor system.

The first five component values of the equilibrium point are given in Table 1, and it can be followed that this equilibrium point is stable as the eigenvalues are inside the unit circle. 

**Figure 4:**
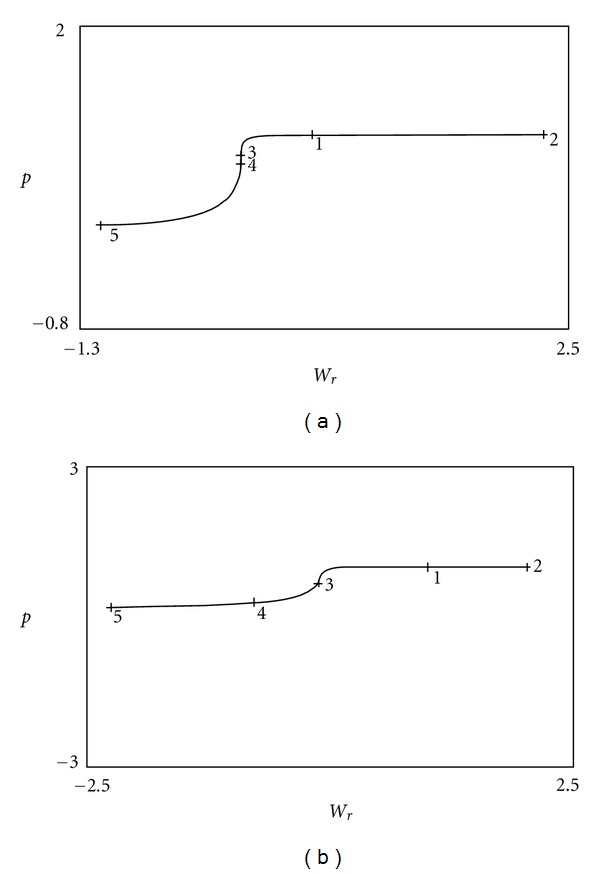
Bifurcation diagram of *p*_pm_ according to *W*_*r*_ parameter. (a) Before learning begins. (b) After learning is completed.

The bifurcation diagram drawn at this point according to *W*_*r*_ parameter is given in Figure 4(a). The labeled points in the diagram show the bifurcation points and are listed in Table 2. At the point with label 4 in the diagram, there is a Hopf bifurcation. The existence of Hopf bifurcation denotes that the system behavior changes with the parameter value. Thus, the system dynamics changes from steady state solution consisting of equilibrium points to a limit cycle behavior. In our model, while stable equilibrium points denote the choices determined by the action selection module, the limit cycle behavior corresponds to seeking an appropriate choice. So, with the given dynamical system for action selection, both of the explore and exploit features of reinforcement learning process can be realized.

The fixed points for the previous parameters are given in Table 3. With the previous parameters, the bifurcation diagram in Figure 4(b) is obtained. The labeled points in the diagram show the bifurcation points and are listed in Table 4. At the points with label 3 and 4 in the diagram, there is a Hopf bifurcation.

After the learning ends, the weight matrices become:

(2) $$\begin{array}{c} W_{r}=\left[\begin{array}{c} 1\\ 0.7018 \end{array}\right],\;\;W_{c}=\left[\begin{array}{cc} 1.1855 & 0.8380\\ 0.2518 & 0.3833 \end{array}\right],\\ W_{v}=\left[\begin{array}{cc} 0.5409 & 0.5409 \end{array}\right]. \end{array}$$

When the bifurcations diagrams of before and after learning are compared, the most important difference is the unstable parameter range. It is larger in the after learning diagram than the before learning diagram. According to the reinforcement learning principle, the system tries actions that it has not selected before to discover actions that effectively produce reward. This trial phase is explained by the term exploration. This exploration phase in reinforcement learning corresponds in a way to deliberately choosing actions currently estimated to be suboptimal in order to reduce uncertainty about them in neuroscience jargon. The parameter values corresponding to unstable region and Hopf bifurcation evoke the “exploration” process of reinforcement learning, while the parameter values giving rise to stable equilibrium points correspond to an action selected. Therefore, the bifurcation diagrams show that the A-S module is capable of revealing two different types of behaviors (two stable equilibrium points), as well as the case where learning does not take place and the system is indecisive (unstable equilibrium point). 

### 4.3. Implementation of Reinforcement Learning in the Model

The DA signaling module uses reinforcement learning to effect the next step decisions of the A-S module. As in *‎*[11], the effect of DA is demonstrated by a difference equation in order to model the dynamic behavior of the process:

(3) $$\begin{array}{c} u_{\text{DA}}\left(k+1\right)=u_{\text{DA}}\left(k\right)+mu_{\text{DA}}\left(-u_{\text{DA}}\left(k\right)+s_{\text{DA}}\left(ri,Ni\right)\right). \end{array}$$

The activation function *s*_DA_ is a sigmoidal function given as

(4) $$\begin{array}{c} s_{\text{DA}}\left(ri,Ni\right)=0.5\cdot \left(1+\tanh\left(Ni\cdot ri-\theta_{\text{DA}}\right)\right). \end{array}$$

The nAchR activity is modeled by three dynamical variables as in Appendix A. *Ni* is the upregulation of nAchRs by nicotine and is represented by the product of the values of n and s signals when nicotine injection stops (Appendix A). With nicotine present in the environment, the nAchR activation increases. However, opponency (brain homeostatic system) acts to normalize this activity to normal levels, forming the opponent-process mechanism in this system. *θ*_DA_ is the threshold setting the minimum tonic DA level. We take *θ*_DA_ = 0.01. *ri* is the reward signal initiated by nicotine taking. *mu*_DA_ is the learning rate in the DA subsystem.

Previous works by [39–42] suggest action selection models for the cortico-basal ganglia-thalamic loop. In our action-selection module which is acquired from *‎*[12], there are two components: premotor and motor loops which model the dynamical system of cortex-basal ganglia-thalamus (C-BG-TH) loops. The relevant equations for premotor and motor loops are given in Appendix B.

*Wd*_pm/m_ adds the diffusive effect of subthalamic nucleus and is a symmetrical matrix. The diagonal matrix *Wr*_pm_ represents the effect of ventral striatum (nucleus accumbens) on dorsal striatum (caudate nucleus and putamen). Ventral pathway is effective in evaluation while dorsal pathway is responsible for goal-directed behavior. Therefore, *Wr*_pm_ parameter shows the modulation of action choices according to evaluation. *Wr*_pm_ parameter is modified according to reinforcement learning, and by changing *Wr*_pm_, the selected actions can be changed. The representation of sensory stimulus is formed by the matrix *Wc*_pm_. As explained in Section 2.1, previous actions impact the current reward value and therefore modulate the current action choice. The adaptation of weights *Wc*_pm_ and *Wr*_pm_ is done as follows:

(5) Wcpm(k+1)=Wcpm(k)+ηc·δ(k)·pm(k)·I(k)′,Wrpm(k+1)=Wrpm(k)+ηr((U−DA+Ni)(uDA(k)−θwDA)′   ×(pm(k)−θ))′·f(pm(k))·rm(k).

*Wr*_pm_ is calculated only after the reward signal *r*_*i*_ becomes greater than 0.5. This is done because in real life, at first there is no powerful fondness emotion for cigarettes. After the person has started to smoke more frequently than casually, the reward value of smoking becomes greater and modulatory. Therefore, in the model, we did not take reward into account until it passes a threshold and becomes modulatory.

${\overset{-}{U}}_{\text{DA}}$ is the running average of 10 steps denoted as in *‎*[11]. Thresholds for *U*_DA_ and *p*_*m*_, respectively, are *θ*_DA_ and **θ**, and are taken as 0.1 times their respective signal. The learning rate *η* is taken as 0.1. The variable *δ* represents the error in expectation and is calculated as

(6) $$\begin{array}{c} \delta \left(k\right)=ri+\mu V\left(K+1\right)-V\left(k\right). \end{array}$$

The evaluation of the action selection based on the sensory input to cortex denoted by *I*(*k*) and the corresponding reward is given as the value signal:

(7) $$\begin{array}{c} V\left(k\right)=\left(W\nu +\text{base}\right)I\left(k\right). \end{array}$$

Here, *W*_*v*_ is a row vector and the term *base* is a row vector with identical entries. An expectation signal based on the value signal is generated which, together with *r*_*i*_, gives rise to the error *δ*. The error signal represents the modulating role of the neurotransmitters and modulates the behavior of dorsal striatum stream via *Wr*_pm_. The error signal strengthens the representation of the sensory input *I*(*k*) via *Wc*_pm_ and updates the value of stimuli via *W*_*v*_ as follows:

(8) $$\begin{array}{c} W\nu \left(k+1\right)=W\nu \left(k\right)+\eta_{\nu }\delta \left(k\right)I{\left(k\right)}^{\prime }. \end{array}$$

## 5. Results

To measure the performance of the proposed model, the response to nicotine-taking explained in *‎*[11] is considered. At the beginning, the reward value (*r*_*i*_) is very small (like 0.01). Each time the selected action is smoking, *r*_*i*_ is multiplied by 2 until *r*_*i*_ = 1. After 20 successive smoking decisions, the system is considered to become the model of an addict. 20 is not a magic number, but a rather statistical border we applied. As seen from the inclination of the *Wr*_pm_ values in Figure 6, when a behavior is learned (or stamped in since this is an addiction), the behavior is not modified in the future. So, a limit number of 20 selections is sufficient to demonstrate the case. 

**Figure 5:**
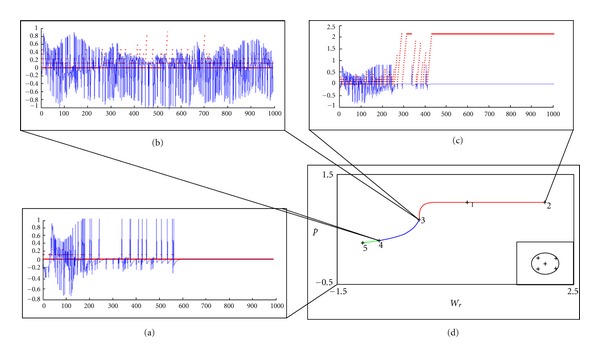
Error in expectation for each state of the system. In (a), (b), and (c), the *δ* error signal in expectation (blue lines) and smoking actions (red dots) are drawn. (a) Initially, the system randomly selects smoking or no smoking, but as time goes by, the system learns to select no smoking action. When no smoking behavior is firmly adopted, *δ* becomes 0 and no smoking action is always selected. (b) No particular behavior is adopted. The *δ* error signal oscillates between −1 and 1 throughout the simulation period, and the system randomly selects smoking or no smoking. The system does not learn any behaviors. (c) At first, the system randomly selects smoking or no smoking, but eventually, the system learns to select smoking action repeatedly. Even though no smoking is selected once in a while, the system quickly overcomes this decision and the learned behavior is adopted again. When addiction develops, *δ* becomes 0 and smoking action is always selected. (d) Bifurcation diagram of the action selection system (the branch colors are green, stable branch of nonaddictive behavior; blue, unstable branch of corresponding to exploration; red, stable branch of addiction).

The action selected by the A-S module is determined by calculating the solution of *p*_m_. The value function and the error function given by (6)–(8) are calculated, and using these results the weight matrices *Wc*_pm_, *Wr*_pm_, and *Wv* are updated according to (5) and (8). The simulation stops if the smoking action is selected successively for 20 times in a given time frame which is taken as 1000 steps. If the number of the successive smoking actions does not reach to 20 in the given time interval, the system is considered to model a subject that does not become an addict but a person who, if smokes, smokes only occasionally. The graphs in Figures 5(a), 5(b) and 5(c) reveal that the behavior does not change after it is learned, and a statistical approach to end the simulation at 1000 steps is sufficient to see the results. 

The parameter values used in the simulation are *λ* = 0.5, *β* = 0.03, *a* = 3, *mu*_DA_ = 0.1, *η*_*c*_ = 0.1, *η*_*v*_ = 0.1, and *η*_*r*_ = 0.1, and base is 0.2. The initial values of the weight matrices *Wc* and *Wv* are generated randomly with small positive real numbers. The *Wr*_pm_ matrix is diagonal with all the main diagonal entries having the initial values equal to one. During the updating phase the matrix values *Wc*_pm_ and *Wr*_pm_ are normalized. The matrices *Wd*_pm/m_ and *Wr*_m_ are composed of 0.5's and they are constant. The noise signal is generated as a very small random number. The action outputs are coded as [1 0]′ for smoking, [0 1]′ for nonsmoking, and [1 1]′ for indecisive behaviors.

In 50 successive runs, the model completed the task of becoming an addict in 22 of the simulations on average 363 steps out of 1000 steps with a standard deviation of 288.5952. The final matrices for a trial when addiction is set up are given as follows:

(9) $$\begin{array}{c} Wr_{\text{pm}}=\left[\begin{array}{c} 1\\ 0.6179 \end{array}\right],\;\;Wc_{\text{pm}}=\left[\begin{array}{cc} 0.8569 & 0.2965\\ 0.16 & -0.4331 \end{array}\right]. \end{array}$$

The graphs of the expectation errors for each state of the system are given in Figure 5. In Figures 5(a), 5(b), and 5(c), the *δ* error signal in expectation (blue lines) and smoking actions (red dots) are drawn. Each red dot shows the action choice, being 0 when no smoking is selected or a positive decimal number showing the number of successive smoking actions. The *y*-axis is normalized by 10 for graphical reasons (e.g., when *y* = 2, it is actually 20. When no smoking action is selected, *y*-axis is 0. If the current action is the 5th smoking action selected in a row, then *y* = 0.5). We accept that addiction develops when smoking is successively selected for 20 times. Error signal *δ* remains constant if the same choice is made successively, and changes otherwise. In (a), initially the system randomly selects smoking or no smoking, but as time goes by, the system learns to select no smoking action repeatedly. When no smoking behavior is firmly adopted, *δ* becomes 0 and no smoking action is always selected. Figure 5(b) shows that the *δ* error signal oscillates between −1 and 1 throughout the simulation period, and the system randomly selects smoking or no smoking. The system does not learn any behaviors, namely it learns to be indecisive about smoking. In (c), at first the system randomly selects smoking or no smoking, but as time goes by, the system learns to select smoking action repeatedly. Even though no smoking is selected once in a while, the system quickly overcomes this decision and the learned behavior is adopted again. When addiction develops, *δ* becomes 0 and smoking action is always selected. Figure 5(d) shows the bifurcation diagram of the system with each branch corresponding to a different behavioral pattern colored differently.

**Figure 6:**
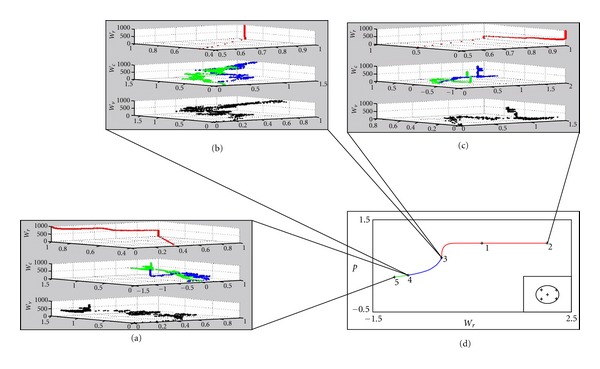
Change in *W* parameters in each state of the system. (a) Addiction does not develop. After the learning ends, *Wr* values shift to [0 1], namely which is the code of nonsmoking action in our simulation. (b) At the end of the simulation period, *Wr* values are shifted to [1 1]. No particular behavior is adopted. (c) When learning ends and addiction develops, *Wr* values shift to [1 0], which represents the code for smoking action in the simulation. (d) Bifurcation diagram of the action selection system (already given in Figure 4) (the branch colors are green, stable branch of nonaddictive behavior; blue, unstable branch corresponding to exploration; red, stable branch of addiction).

The graphs of the change in W parameters in the previously mentioned simulations are given in Figure 6. *Wc* shows the association of the environmental effects with the current actions. In (a), after the learning ends, *Wr* values shift to [0 1], namely which is the code of “nonsmoking action” in our simulation. In (b), at the end of the simulation period *Wr* values are shifted to [1 1], namely no particular behavior is adopted. In (c), when learning ends and addiction develops, *Wr* values shift to [1 0], which represents the code for smoking action in the simulation. Figure 6(d) shows the colored bifurcation diagram of the system. Notice that even the opposite of the adopted action is selected once in a while, the graph of *Wr* parameter does not change its inclination. For example in Figure 5(c), at about 300 steps, the system selects smoking for some time, but then it skips and selects no smoking. At the corresponding steps in Figure 6(c), we can see that *Wr* has already shifted to [1 0] corner of the graph, and it does not change its direction even though no smoking action is selected for a couple of times. This proves that the system learns to select smoking behavior. After about 400 steps, the system always selects smoking action.

## 6. Conclusion and Discussion 

According to our hypothesis, nicotine addiction is a transition from impulsive behavior to compulsive behavior developed through reinforcement learning. Reward evaluation in the brain is one of the key processes that limbic system elements take place, and it can be stimulated easily by reinforcement learning tasks. Thus, our reinforcement learning-based action selection model enables us to understand and follow the ongoing behavioral modification processes in brain subsystems.

DA is used as the neuromodulatory element of this model. The DA is secreted by the VTA, stimulates the action selection task in the basal ganglia, and has role in coding the learned behaviors. DA transmission between the cortical layers (PFC, OFC, and mOFC) and the amygdala and hippocampus is effective in learning. DA secretion in the NAc is used in evaluating the behaviors and coding the reward values. DA projections in the basal ganglia structures are used for decision processes. Knowing that DA has so many different roles in the brain, the proposed model decreases these areas to a single type of DA transmission in order to simplify the modeling.

Brain process modeling can be handled in several levels such as system level, single nucleus function level, and molecular level. Our proposed model utilizes a combination of these different approaches. To model the effect of nAchRs in modulating, the DA secretion from the VTA provides a quite realistic approach to handle the reward evaluation. The action selection module of the model is based on a system level approach, and each equation written for each component of this module corresponds to a single basal ganglia nucleus. Since the model is composed of different blocks, taking each block as a submodel and connecting these submodels using input-output relationships justifies the different modeling approaches we used.

The proposed model demonstrates that nicotine addiction is a type of learned behavior, and the process that leads an individual to become an addict can be explained as malfunctioning of goal-directed behavior. So, the interaction of the corticostriatothalamic action selection loop with the upper cortical parts for learning and lower limbic components for reward evaluation is considered. It is discussed that these parts of brain circuitry have a role in the development of addiction. It is demonstrated with the model that cumulative effects of the previous actions are stored in memory and are used to evaluate the possible outcomes of the present actions. 

This work represents progress towards a unified computational model of the addiction development process in the brain. Furthermore, this paper is a complete presentation of the work in [13, 14]. The sources of DA secretion and different roles of DA in the brain remain as the improvement areas of the model. The action selection loop in the model is triggered by input to the PFC. However, striatum is known as the modulatory input nucleus of basal ganglia, so the action selection subsystem dynamics needs to be improved to accept modulatory input from the striatum. The inner reward value in our model is geometrically increasing. However, the evaluation of the reward in the mesolimbic system should be considered as another dynamic system modulated by the action outcomes which must be taken into account by future work. We emphasize reinforcement learning as the major process underlying addiction; however, future work should also consider other psychological processes and the relevant neurotransmitters such as glutamate and GABA as well.

### 6.1. The Difference and Improvements Brought by Our Model

Our work proposes a cortico-striato-thalamic A-S circuit driven by the effects of nicotine-taking as a model for nicotine addiction. The A-S circuit has two parts, an action selection part corresponding to the dorsal stream which simulates behavioral choices, and a second part corresponding to the ventral stream which simulates the evaluation of the action choices and modulates the action selection. The A-S circuit utilizes competitive learning which is modified with the VTA DA signaling affected by the nicotine. While the structure of the A-S circuit is an interconnected nonlinear dynamical system corresponding to premotor and motor loops of the PFC-basal ganglia-thalamus, the modifications of the action choices are realized by changing a parameter in the premotor loop. This parameter, Wr, corresponds to the modulatory effect of DA through reinforcement learning process. By adapting the *Wr* parameter, the system can learn to be an addict, a nonaddict, and an indecisive person who adopts neither of the behavioral patterns. Thus, the A-S module, unlike the one in *‎*[11], is capable of revealing reinforcement learning. In *‎*[11], reinforcement learning is mentioned; however, the model does not administer nicotine-taking through its own choices. The dynamical systems considered are represented by nonlinear discrete time systems, so while the model projects the nonlinearity and dynamics of the cognitive processes emerging, it is still easy to follow the dynamic behavior emerged with parameter changes.

The aim in this paper is to support the idea that addiction develops as a form of goal-directed behavior, and therefore, the interaction of cortico-striato-thalamic action selection loops has an important role in the development of addiction. Since the cumulative effects of the previous actions trigger the present action selection, this mechanism is modeled as a nonlinear dynamical system to realistically simulate the addiction process and the role of reinforcement learning in neural structures. 

Furthermore, we improved the n-s-c circuit given in *‎*[11] to demonstrate the change in the reaction of the nAchRs against blood nicotine level when addiction develops. By modifying the activation functions parametrically (Appendix A), the disruptive effect of the addiction on opponent-process can be observed. The values of the *γ* and *φ* parameters are changed in the scope of [0.8,1] to obtain the graph given in Figure 1.

## Supplementary Material

The simulation results given in the paper are obtained by the m-files for the model behavior and by XPPAUT codes for bifurcation diagrams. Both of these source codes are provided as supplementary material and can be downloaded from http://www.selinmetin.info/.

## Figures and Tables

**Table 1:** The fixed points obtained before the weight matrices are adjusted by learning. In the right columns, the eigenvalues of the linearized system at these fixed points are shown.

	Equilibrium point	Eigenvalues				
*p*_pm_	0.9982	0.06	−0.05	0 + *i* 0.02	0 − *i* 0.02	0

**Table 2:** Bifurcation values of *p*_pm_ according to *W*_*r*_ parameter before learning.

TY	LAB	*W*_*r*_	*p*_pm1_
EP	1	0.5	0.9981
EP	2	2.32	0.9983
LP	3	−0.046	0.816
HB	4	−0.045	0.735
EP	5	−2.24	0.133

(TY: type of bifurcation, LAB: label, EP: end point, LP: limit point bifurcation, and HB: hopf bifurcation).

**Table 3:** The fixed points obtained after the weight matrices are adjusted by learning. In the right columns the eigenvalues of the linearized system at this fixed point are shown.

	Equilibrium point	Eigenvalues				
*p*_pm1_	0.9989	0.05	−0.05	0 + *i* 0.04	0 − *i* 0.035	0

**Table 4:** Bifurcation values of *p*_pm_ according to *W*_*r*_ parameter after learning.

TY	LAB	*W*_*r*_	*p*_pm1_
EP	1	1	0.9989
EP	2	2.03	0.9989
HB	3	−0.116	0.695
HB	4	−0.782	0.298
EP	5	−2.247	0.211

(TY: type of bifurcation, LAB: label, EP: end point, LP: limit point bifurcation, and HB: hopf bifurcation).

## Appendices

### A.

With chronic nicotine consumption, nAchRs on DA neurons act in three different time scales. The rapid DA signal increase is in parallel with the nAchR response, *n*. Upregulation is an increase in the number of nAchRs on the surface of the DA neurons which makes them more sensitive to nicotine. *s* signal shows the upregulation of the nAchRs. The long-term opponency in the brain which tries to bring nAchR response to normal level is denoted by *c*. Of these signals, *n* is the fastest and *c* is the slowest [11].

The initial values of *n*, *s*, and *c* are

(A.1) $$\begin{array}{c} n=0.2,\;\;s=0.2,\;\;c=0.2. \end{array}$$

The parameters used in the equations are

(A.2) $$\begin{array}{c} \theta_{n}=0.6,\;\;\theta_{s}=0.7,\;\;\theta_{c}=0.7,\;\;\beta_{s}=0.4,\\ \beta_{c}=0.4,\;\;\text{nicotine}=\{\begin{array}{cc} 0.3, & k<250\\ 0, & \text{else}. \end{array} \end{array}$$

The rates used in equations are

(A.3) $$\begin{array}{c} \mu =0.1,\;\;\tau_{n}=0.25,\;\;\tau_{s}=1,\;\;\tau_{c}=2. \end{array}$$

The activation functions are

(A.4) $$\begin{array}{c} \alpha_{n}\left(k\right)=0.5\cdot \left(1+\tanh\left(\text{nicotine}-\gamma_{n}\cdot \theta_{n}\right)\right),\\ \alpha_{s}\left(k\right)=0.5\cdot \left(1+\tanh\left(n\left(k\right)-\gamma_{s}\cdot \theta_{s}\right)\right),\\ \alpha_{c}\left(k\right)=0.5\cdot \left(1+\tanh\left(s\left(k\right)-\gamma_{c}\cdot \theta_{c}\right)\right),\\ \beta_{n}\left(k\right)=0.5\cdot \left(1+\tanh\left(\varphi_{n}\cdot c\left(k\right)-\gamma_{n}\cdot \theta_{n}\right)\right). \end{array}$$

By changing the values of the *γ* and *φ* parameters in the scope of [0.8,1], the effect of becoming an addict on the opponent-process can be demonstrated as in Figure 1. In addicts, the B-process lasts longer, and the neutral levels of the opponent-processes are lower than the neutral levels in healthy people.

The dynamic equations of *n*, *s*, and *c* are

(A.5) $$\begin{array}{c} n\left(k+1\right)=n\left(k\right)+\frac{\mu }{\tau_{n}}\left[-\beta_{n}\left(k\right)\cdot c\left(k\right)+\alpha_{n}\left(k\right)\cdot \left(1-n\left(k\right)\cdot c\left(k\right)\right)\right],\\ s\left(k+1\right)=s\left(k\right)+\frac{\mu }{\tau_{s}}\left[-\beta_{s}\cdot s\left(k\right)+\alpha_{s}\left(k\right)\cdot \left(1-s\left(k\right)\right)\right],\\ c\left(k+1\right)=c\left(k\right)+\frac{\mu }{\tau_{c}}\left[-\beta_{c}\cdot c\left(k\right)+\alpha_{c}\left(k\right)\cdot \left(1-c\left(k\right)\right)\right]. \end{array}$$

### B.

In our action selection module which is acquired from [12], there are two components: premotor and motor loops. The equations for premotor (pm) and motor (*m*) loops of the cortex-basal ganglia-thalamus (C-BG-TH) dynamical system in the A-S module are given as follows:

(B.1) $$\begin{array}{c} p_{\text{pm}}\left(k+1\right)=f\left(\lambda p_{\text{pm}}\left(k\right)+m_{\text{pm}}\left(k\right)+Wc_{\text{pm}}I\left(k\right)\right),\\ m_{\text{pm}}\left(k+1\right)=f\left(p_{\text{pm}}\left(k\right)-d_{\text{pm}}\left(k\right)\right),\\ r_{\text{pm}}\left(k+1\right)=Wr_{\text{pm}}f\left(p_{\text{pm}}\left(k\right)\right),\\ n_{\text{pm}}\left(k+1\right)=f\left(p_{\text{pm}}\left(k\right)\right),\\ d_{\text{pm}}\left(k+1\right)=f\left(Wd_{\text{pm}}n_{\text{pm}}\left(k\right)-r_{\text{pm}}\left(k\right)\right),\\ p_{\text{m}}\left(k+1\right)=f\left(\lambda p_{\text{m}}\left(k\right)+m_{\text{m}}\left(k\right)+\beta p_{\text{pm}}\left(k\right)+\text{noise}\right),\\ m_{\text{m}}\left(k+1\right)=f\left(p_{\text{m}}\left(k\right)-d_{\text{m}}\left(k\right)\right),\\ r_{\text{m}}\left(k+1\right)=Wr_{\text{m}}f\left(p_{\text{m}}\left(k\right)\right),\\ n_{\text{m}}\left(k+1\right)=f\left(p_{\text{m}}\left(k\right)\right),\\ d_{\text{m}}\left(k+1\right)=f\left(Wd_{\text{m}}n_{\text{m}}\left(k\right)-r_{\text{m}}\left(k\right)\right). \end{array}$$

The variables *p*_pm/m_, *m*_pm/m_, *r*_pm/m_, *n*_pm/m_, and *d*_pm/m_ stand for vectors corresponding to cortex, Thl, Str, STN, and GPi/SNr constituents of premotor and motor loops, respectively. The dimensions of these vectors are determined by the number of action choices. For nicotine addiction, two actions are considered, “smoke” and “not smoke”, so the dimension of the system as a whole is 20. Just like in *‎*[12], the action selection module decides on an action outcome by evaluating the value of the presented stimuli. However, if the reward signal generated for that stimulus is disappointing, a random response is generated. To enable randomness, a noise signal is added to the motor loop. The premotor part completes the evaluation and determines possible actions, and then the motor part decides on one of these possibilities by acting as a fine discriminator. The sensory stimulus denoted by *I* affects the premotor loop, and the output of this loop modulates the motor loop, resulting in the action. For nicotine addiction model at this level, this sensory stimulus is considered to be neutral and a 2-dimensional vector is used with same small component values.

The nonlinear function is sigmoid and given as

(B.2) $$\begin{array}{c} f=0.5\left(1+\tanh\left(a\left(x-0.45\right)\right)\right). \end{array}$$
